# Impact of Changes in Perinatal Care on Neonatal Respiratory Outcome and Survival of Preterm Newborns: An Overview of 15 Years

**DOI:** 10.1155/2012/643246

**Published:** 2012-12-20

**Authors:** Filipa Flor-de-Lima, Gustavo Rocha, Hercília Guimarães

**Affiliations:** ^1^Neonatal Intensive Care Unit, Pediatric Integrated Hospital, São João Hospital, Alameda Professor Hernâni Monteiro, 4200-319 Porto, Portugal; ^2^Faculty of Medicine, Porto University, Alameda Professor Hernâni Monteiro, 4200-319 Porto, Portugal

## Abstract

Survival and outcomes for preterm infants with respiratory distress syndrome (RDS) have improved over the past 30 years. We conducted a study to assess the changes in perinatal care and delivery room management and their impact on respiratory outcome of very low birth weight newborns, over the last 15 years. A comparison between two epochs was performed, the periods before and after 2005, when early nasal continuous positive airway pressure (NCPAP) and Intubation-SURfactant-Extubation (INSURE) were introduced in our center. Three hundred ninety-five clinical records were assessed, 198 (50.1%) females, gestational age 29.1 weeks (22–36), and birth weight 1130 g (360–1498). RDS was diagnosed in 247 (62.5%) newborns and exogenous surfactant was administered to 217 (54.9%). Thirty-three (8.4%) developed bronchopulmonary dysplasia (BPD), and 92 (23%) were deceased. With the introduction of early NCPAP and INSURE, there was a decrease on the endotracheal intubation need and invasive ventilation (*P* < 0.0001), oxygen therapy (*P* = 0.002), and mortality (*P* < 0.0001). The multivariate model revealed a nonsignificant reduction in BPD between the two epochs (OR = 0.86; 95% CI 0.074–9.95; *P* = 0.9). The changes in perinatal care over the last 15 years were associated to an improvement of respiratory outcome and survival, despite a nonsignificant decrease in BPD rate.

## 1. Introduction

Respiratory distress syndrome (RDS), caused mainly by lung immaturity and surfactant deficiency [1] contributes to significant morbidity and some mortality in very low birth weight (VLBW) preterm newborns [2]. Changes in perinatal care, such as the use of antenatal steroids, exogenous surfactant administration, early nasal continuous positive airway pressure (NCPAP), and lung protective strategies of mechanical ventilation (patient-triggered modalities, volume controlled modes and high frequency oscillatory ventilation), have led to an improvement of survival and outcomes of infants with RDS over the past 30 years [1]. 

Exogenous surfactant reduces mortality and short-term respiratory morbidity in premature infants with RDS [3]; however, intubation and invasive mechanical ventilation are often required for its administration. In addition, prolonged invasive ventilation increases the risk of subsequent bronchopulmonary dysplasia (BPD) [4]. Early treatment with NCPAP helps the very preterm to rapidly achieve the functional residual capacity, stabilize the thoracic cage and airways, preserve endogenous surfactant, and reduce the need for invasive ventilation, and exogenous surfactant administration [5–8], but it may be an insufficient respiratory support for a significant number of infants born before 26 weeks of gestation [9]. Although there is a growing body of evidence to guide decision making, there is, not yet, consensus on the best treatment approach for acute RDS [1]. 

The aims of our study were to assess the changes in perinatal practices for VLBW preterm newborns admitted to our neonatal intensive care unit (NICU) over the last 15 years and to perform a comparison of respiratory outcome between two epochs, the periods before 2005 and after, when early NCPAP and Intubation-SURfactant-Extubation (INSURE) were introduced in our center.

## 2. Material and Methods

A retrospective study, from 1997 to 2011, was performed at our center, a level III NICU, referral center for cardiac and surgical patients for the north of Portugal, with an average of 450 admissions per year, including about 50 VLBW infants. Preterms with birth weight less than 1500 g were included in the study. Newborns affected of major congenital anomalies, a TORCH infection, hydrops fetalis, and chromosomal anomalies, as well as the outborns and those transferred to other NICUs before 36 weeks of corrected gestational age, were excluded. 

Clinical records were reviewed; demographics, histological chorioamnionitis and clinical data were assessed: gender, gestational age, birth weight, antenatal steroids pulses, delivery mode, respiratory support in the delivery room, Apgar score, the presence of RDS, the need for exogenous surfactant administration and mode of administration; respiratory support in the NICU, the need for oxygen therapy, the prevalence of BPD and other major morbidities (nosocomial sepsis, necrotizing enterocolitis, severe intraventricular hemorrhage, retinopathy of prematurity, patent ductus arteriosus, periventricular leukomalacia), and length of NICU stay and survival. 

Antenatal steroid regimen was performed with dexamethasone (total dose of 24 mg, divided into two doses given intramuscularly every 12 hours) until 2003, and with betamethasone (24 mg, divided into two doses given intramuscularly 24 hours apart) thereafter, in pregnancies with threatened preterm labour below 35 weeks gestation.

Gestational age (in this study we considered the completed weeks) was assessed by menstrual age (women with regular menstrual cycles), ultrasound examination (when a discrepancy of two or more weeks existed between the age derived by menstrual dating and the age derived sonographically, or in the absence of a menstrual date) [10], or the New Ballard Score (in the absence of obstetrical indexes) [11]. Small for gestational age was defined as a birth weight below 10th centile of Lubchenco's fetal growth charts before 2003 [12] and a birth weight below 10th centile of Fenton's fetal growth charts after 2003 [13]. 

Early NCPAP is usually started immediately (first 15 minutes) after birth, although, in this study, it was considered when started in the first 30 minutes of life, once some patients were transported to the NICU, monitorized, and in spontaneous breathing. The first and 5th minute Apgar scores were dichotomized in two groups (≤7 and ≥8). RDS diagnosis was made on a combination of clinical and radiographic features according to the criteria of RDS of the Vermont Oxford Network: (1) PaO_2_ < 50 mmHg in room air, central cyanosis in room air, a requirement for supplemental oxygen to maintain PaO_2_ > 50 mmHg or a requirement for supplemental oxygen to maintain a pulse oximeter saturation over 85% within the first 24 h of life; and (2) a chest radiograph consistent with RDS (reticulogranular appearance to lung fields with or without low lung volumes and air bronchograms) within the first 24 h of life. The diagnosis of BPD was made in preterm newborns with gestational age 32 weeks or below, if the infant was chronically oxygen dependent at 36 weeks of corrected age and had a characteristic chest radiograph. In newborns above 32 weeks gestational age, BPD was considered if the baby was dependent on oxygen for 28 consecutive days [14]. Oxygen was used to maintain saturations given by pulse oximetry in the range of 88 to 94% for RDS and 90 to 95% for BPD. Exogenous surfactant was administered through the endotracheal tube in babies on invasive mechanical ventilation or by INSURE in babies off invasive mechanical ventilation [15]. 

Routine mechanical ventilation modes were patient-triggered modalities using a Babylog 8000+ (*Drager, Lubeck, Germany*), SIPPV (synchronized intermittent positive pressure ventilation) until 2000, and SIPPV + VG (volume guarantee) or PSV + VG (pressure support ventilation + volume guarantee) after 2000 [16]. High frequency oscillatory ventilation, at our unit, is used as a rescue ventilation using the Sensor Medics 3100 A (Sensor Medics Corporation, Yorba, Linda, CA, USA). Not intubated patients were placed on *InfantFlow* nasal CPAP (Care Fusion, Yorba Linda, USA) with nasal prongs or mask with a pressure of 5–7 cmH_2_O, in prone position and started on caffeine, kept until 34 weeks of gestational age. Starting total fluid intake was 70 mL/kg/day and increased daily according to the hemodynamic status. Nasal CPAP (*InfantFlow*) was used with pressures of 5-6 cmH_2_O in most cases but could be increased up to 7-8 in particular cases. Patients with apnoeas requiring stimulation were changed to NCPAP with synchronized pressure assistance (Infant Flow SiPAP, Viasys Health Care, Palm Spring, USA). Patients requiring FiO_2_ > 0.40 with respiratory distress and/or arterial PCO_2_ > 65 mmHg and pH < 7.20 were intubated for exogenous surfactant administration (poractant alfa). INSURE was routinely performed after an intravenous bolus of morphine (0.1 mg/kg). Naloxone (0.1 mg/kg, IV push) was used if needed, to reverse respiratory depression caused by morphine.

As early NCPAP and INSURE were introduced in our NICU in 2005, we compared demographic and clinical characteristics between two epochs (1997–2004 and 2005–2011, before and after their introduction, resp.). We, also, compared demographic and clinical characteristics between surviving preterm with and without BPD.

Histological chorioamnionitis was defined according to the Blanc classification [17]: stage I, intervillositis; stage II, chorionitis; stage III, chorioamnionitis; funisitis, polymorphonuclear leukocytes in the Wharton's jelly or umbilical vessel walls; vasculitis-polymorphonuclear leukocytes in chorionic or umbilical blood vessel walls. All stages of chorioamnionitis were considered together. Proven neonatal sepsis was defined as any systemic bacterial or fungal infection documented by a positive blood culture. The criteria of Bell were used for the diagnosis and staging of necrotizing enterocolitis [18]. Staging of retinopathy of prematurity was done according to the International Classification [19, 20]. Intraventricular haemorrhage was classified according to Papile et al. [21]. Periventricular leukomalacia was classified according to de Vries and Rennie [22]. Hemodynamically significant patent ductus arteriosus was diagnosed on the basis of the echocardiographic findings. The first evaluation is usually between 24 and 72 hours of life, with daily evaluation until closure of the ductus. The standard treatment is indomethacin. 

The study protocol has been approved by our institute's committee on human research. 

Continuous variables with symmetric distribution were characterized by mean (± standard deviation), those with asymmetric distribution by median (minimum–maximum values) and categorical variables were characterized by its absolute and relative frequencies. Mann-Whitney *U* test was used to compare two independent samples (asymmetric continuous variables) and chi-squared test or Fisher's exact test to compare categorical variables, the latest one for contingency tables 2 × 2 when expected values were less than 5. A multivariate analysis by logistic regression was performed to evaluate the outcome BPD. The results are presented by odds ratio (OR), 95% confidence interval (CI), and *P* value. The statistical analysis was performed using SPSS program v.19 (IBM, New York, USA) and a *P* value <0.05 was considered significant. 

## 3. Results 

Out of 735 clinical records, 395 were reviewed, with 198 (50.1%) females, mean gestational age 29.1 weeks (22–36), birth weight 1130 g (360–1498), and 95 (24.1%) small for gestational age. Three hundred and forty patients were excluded (outborns = 80, transferred before 36 weeks gestational age = 201, major congenital anomalies = 25, TORCH infection = 29, hydrops fetalis = 3, chromosomal anomalies = 2).

During the antenatal period, dexamethasone was used in 161 (49.5%) newborns and betamethasone in 164 (50.5%), with a full cycle in 213 (65.5%) cases (Table 1). The overall delivery room data and major morbidity are reported on Table 1. 

Comparing both epochs (1997–2004 and 2005–2011), male gender was predominant (60% versus 39.5%) before 2005 (*P* < 0.0001). Betamethasone was administered to 163 (94.8%) newborns (*P* < 0.0001) and 139 (80.8%) had a full antenatal steroid cycle (*P* < 0.0001) after 2005. Also C-section was higher (69.2% versus 56.5%, *P* = 0.009) after 2005. After 2005, the need for endotracheal intubation decreased from 75% to 40.5% (*P* < 0.0001), the prevalence of RDS decreased from 66% to 59% (*P* = 0.15), the need of one single dose of surfactant increased from 27.7% to 48.6% (*P* = 0.001), and the need of two doses of surfactant decreased from 59.8% to 40% (*P* = 0.001). There was a delay in surfactant administration from 1–3 hours to 1–16 hours in the first dose (*P* < 0.0001) and from 6–16 hours to 3–96 hours in the second dose (*P* = 0.03). The need for invasive mechanical ventilation (*P* < 0.0001) and oxygen therapy (*P* = 0.002) decreased, along with a significant improvement of outcomes, less BPD (*P* = 0.022) and mortality (*P* < 0.0001) (Table 1). The causes of death were extreme immaturity/RDS (*n* = 30), sepsis (*n* = 33), severe intraventricular haemorrhage (*n* = 18), necrotizing enterocolitis (*n* = 5), and others (*n* = 5).

Demographics and clinical characteristics of surviving BPD patients are reported in Table 2. 

The multivariate logistic regression, adjusted for gestational age, birth weight, complete antenatal steroid cycle, male gender, histological chorioamnionitis, early NCPAP use, 5th Apgar score, RDS, surfactant administration, patent ductus arteriosus, and sepsis, revealed a nonsignificant reduction in BPD between the two epochs (OR = 0.86; 95% CI 0.074–9.95; *P* = 0.9). Male gender (OR = 0.5; 95% CI 0.22–1.18; *P* = 0.11) and vaginal delivery (OR = 1.46; 95% CI 0.58–3.7; *P* = 0.42) were not associated to an increased risk for BPD.

## 4. Discussion

BPD affects thousands of preterm infants each year [23] and its cause is multifactorial. The pathogenesis has been linked to genetic background, lung tissue immaturity, baro and volutrauma from mechanical ventilation, oxidant injury, and proinflammatory mediators [4, 24–26]. The use of antenatal corticosteroids, postnatal surfactant therapy, and modern intensive care has modified BPD into a less-severe disease that is characterized by arrested lung development, the new BPD. As ventilator-induced lung injury is a major contributing factor to BPD [23], early respiratory management of preterm infants affects respiratory outcome. 

Over the last 15 years, there were changes in perinatal care and delivery room management in our center. One of the chief advances has been the routine administration of antenatal steroids. In 1995, the National Institutes of Health and the American College of Obstetricians and Gynecologists formally issued a consensus statement advocating the use of antenatal steroids for induction of fetal maturation, establishing it as a standard of care for perinatal management of preterm deliveries 24 to 34 weeks' gestational age, with the goal of reducing the risks for RDS, intraventricular hemorrhage, and neonatal death [27]. According to Lee et al., there were notable trends for a reduced risk for adverse neonatal outcomes associated with betamethasone compared with dexamethasone for intraventricular hemorrhage and severe retinopathy of prematurity. Also, betamethasone reduces the risk for neonatal death [28]. In our study, according to the policy of the obstetrics department, dexamethasone was replaced by betamethasone in 2003 and the number of completed cycles of antenatal steroids increased since 2005. The use of antenatal steroids increased from the first to the second epoch, highlighting better obstetric practices.

Early NCPAP is an important first-line form of respiratory support in newborns and is used as an alternative to intubation and invasive mechanical ventilation in extremely preterm infants [5, 9]. Since its introduction by Keszler [29] in 1971, several studies in animals and human infants NCPAP have shown promising results in terms of reduction of lung injury, need for mechanical ventilation and incidence of BPD [30–32]. Since the introduction of early NCPAP in 2005, at our center, there was a decrease on endotracheal intubation need. The significant change in delivery room management between the two studied epochs was also reflected by better Apgar scores at first and fifth minutes. Not only there was an increase in NCPAP associated to a decrease in invasiveness of ventilation, but there was also a decrease in both, oxygen therapy need and its duration.

Observational and cohort studies have shown that NCPAP followed by intubation, surfactant administration, and mechanical ventilation only if NCPAP failure criteria are reached reduces the need for mechanical ventilation. Furthermore, a reduced incidence of BPD without increased mortality has been reported [8, 33, 34]. Few randomized controlled trials have compared intubation and mechanical ventilation with early NCPAP or different types of NCPAP and did not highlight significant differences [35, 36]. Since the FDA (*Food and Drug Administration*) release of surfactant to treat RDS in 1989 [37], there has been a deeper understanding of surfactant physiology [38], as well as completion of multiple clinical studies to further delineate and refine the use of exogenous surfactant in RDS beyond the established evidence that surfactant administration reduces pneumothorax, pulmonary interstitial emphysema, and the combined outcome of BPD or death in preterm infants with surfactant deficiency [39, 40]. International guidelines have published recommendations on the optimal surfactant replacement strategy. According to van Kaam et al., with the exception of surfactant timing, these guidelines on surfactant replacement therapy seem to be implemented in daily clinical practice in European NICUs [41]. Since 2005, the use of surfactant in our unit was similar to the previous period; however, less doses were needed. Also there was a decrease on endotracheal intubation need, and on the prevalence of RDS and BPD. In addition, the length of invasive ventilation and oxygen therapy decreased. With these practices, the prevalence of BPD decreased as well as the overall mortality. The retrospective nature of this study does not allow reliable data on these figures. Male gender was predominant in the first period and in BPD group, however, when adjusting these variables in a logistic regression model there were no statistically significant association with the development of BPD. There were no differences in the prevalence of histological chorioamnionitis, nosocomial sepsis, necrotizing enterocolitis, retinopathy of prematurity, intraventricular hemorrhage, patent ductus arteriosus requiring surgical closure, and periventricular leukomalacia between the two compared epochs. 

## 5. Conclusions

The changes in perinatal care introduced at our center over the last 15 years were associated to both, an improvement of respiratory outcome and survival. With the introduction of early NCPAP and INSURE there was a decrease on endotracheal intubation need and invasive ventilation and oxygen therapy, along with less mortality, but a nonsignificant reduction in BPD. The need of administration of one single dose of surfactant increased, and the need of two or more doses decreased.

## Figures and Tables

**Table 1:** Demographics and clinical data.

	Total *N* = 395	1997–2004 *n* = 200	2005–2011 *n* = 195	*P*
Gender, *n* (%)				
Male	197 (49.9)	120 (60)	77 (39.5)	<0.0001*
Female	198 (50.1)	80 (40)	118 (60.5)	
Gestational age (weeks), mean (±SD)	29.1 (2.9)	28.9 (3.2)	29.3 (2.6)	0.15^¥^
Birth weight (grams), median (min–max)	1130 (360–1498)	1120 (460–1495)	1130 (360–1498)	0.958^¥^
Small for gestational age, *n* (%)	95 (24.1)	42 (21)	53 (27.2)	0.15*
Less than 1000 g, *n* (%)	156 (40)	83 (41.4)	73 (37.4)	0.409*
Antenatal steroids, *n* (%)	325 (82.3)	153 (76.5)	172 (88.2)	
Dexamethasone	161 (49.5)	152 (99.3)	9 (5.2)	<0.0001*
Betamethasone	164 (50.5)	1 (0.7)	163 (94.8)	
Full cycle	213 (65.5)	74 (48.4)	139 (80.8)	<0.0001*
Incomplete cycle	112 (34.5)	79 (51.6)	33 (19.2)	
Histological chorioamnionitis, *n* (%)	111 (28)	53 (26.5)	58 (29.7)	0.364*
Delivery mode, *n* (%)				
Vaginal	147 (37.2)	87 (43.5)	60 (30.8)	0.009*
C-section	248 (62.8)	113 (56.5)	135 (69.2)	
Apgar score, *n* (%) 1st minute				
≤7	283 (71.6)	170 (85)	113 (57.9)	<0.0001*
≥8	112 (28.4)	30 (15)	82 (42.1)	
5th minute			
≤7	140 (35.4)	92 (46)	48 (24.6)
≥8	225 (64.6)	108 (54)	147 (75.4)
Respiratory management in the delivery room, *n* (%)				
Endotracheal intubation	229 (58)	150 (75)	79 (40.5)	<0.0001*
Spontaneous ventilation	110 (27.8)	50 (25)	60 (30.8)	0.201*
Early NCPAP	56 (14.2)	0	56 (28.7)	<0.0001*
Respiratory distress syndrome, *n* (%)	247 (62.5)	132 (66)	115 (59)	0.15*
Surfactant administration method, *n* (%)	217 (54.9)	112 (56)	105 (53.8)	0.667*
Endotracheal intubation with mechanical ventilation	202 (93)	112 (56)	90 (85.7)	
INSURE	14 (6.5)	0	14 (13.3)	<**0.0001** ^∞^
Both methods	1 (0.5)	0	1 (1)	
Invasive mechanical ventilation, *n* (%)	250 (63.3)	149 (75.3)	101 (51.8)	<0.0001*
Invasive mechanical ventilation, median day (min–max)	6 (1–184)	6 (1–184)	5 (1–140)	0.44^¥^
NCPAP, *n* (%)	221 (55.9)	67 (33.5)	154 (79.4)	<0.0001*
NCPAP, median day (min–max)	12 (1–75)	10 (1–50)	13.5 (1–75)	**0.049** ^¥^
Oxygen, *n* (%)	272 (68.9)	152 (76)	120 (61.5)	0.002*
Oxygen, median day (min–max)	4 (1–184)	7 (1–184)	2 (1–147)	<**0.0001** ^¥^
NICU stay, median day (min–max)	42 (1–184)	39 (1–184)	45 (1–160)	**0.006** ^¥^
Bronchopulmonary dysplasia, *n* (%)	33 (8.4)	23 (11.5)	10 (5.1)	0.022*
Patent ductus arteriosus (PDA), *n* (%)	115 (29.1)	60 (30)	55 (28)	0.340*
Surgical closure of PDA	15 (3.7)	8 (4)	7 ( 3.5)	0.257*
Nosocomial sepsis, *n* (%)	107 (27)	66 (33)	41 (21)	0.089*
Necrotizing enterocolitis ≥ 2A, *n* (%)	13 (3.2)	6 (3.0)	7 (3.5)	0.534*
Retinopathy of prematurity ≥ 2, *n* (%)	15 (3.7)	8 (4)	7 (3.6)	0.257*
Intraventricular hemorrhage III-IV, *n* (%)	22 (5.5)	6 (8.8)	16 (8.2)	0.736*
Cystic periventricular leukomalacia, *n* (%)	36 (9.1)	27 (13.5)	9 (4.6)	0.065*
Deceased, *n* (%)	91 (23)	70 (35)	21 (10.8)	<0.0001*

*Chi-squared test; ^¥^Mann-whitney *U* test; ^∞^Fisher's exact test; SD: standard deviation; NCPAP: nasal continuous positive airway pressure; INSURE: intubation surfactant extubation; nicu: neonatal intensive care unit.

**Table 2:** Demographic and clinical characteristics of the 304 surviving newborns according to BPD.

	BPD *n* = 23	Non-BPD *n* = 281	*P*
Gender, *n* (%)			
Male	17 (73.9)	125 (45.5)	0.007*
Female	6 (26.1)	156 (55.5)	
Gestational age (weeks), median (min–max)	27 (23–31)	30 (24–36)	<**0.0001** ^¥^
Birth weight (grams), median (min–max)	875 (550–1270)	1220 (500–1500)	<**0.0001** ^¥^
Small for gestational age, *n* (%)	2 (8.7)	83 (29.5)	0.05^∞^
Less than 1000 g, *n* (%)	16 (69.6)	67 (23.8)	<**0.0001** ^∞^
Complete cycle of antenatal steroids, *n* (%)	10 (43.5)	177 (63)	0.064*
Histological chorioamnionitis, *n* (%)	3 (13.0)	15 (5.3)	**0.041** ^∞^
Delivery mode, *n* (%)			
Vaginal	11 (47.8)	87 (31)	0.096*
C-section	12 (52.2)	194 (69)	
Respiratory support in the delivery room, *n* (%)			
Endotracheal intubation	22 (95.7)	120 (42.7)	<**0.0001** ^∞^
Spontaneous	0	106 (37.7)	<**0.0001** ^∞^
Early NCPAP	1 (4.3)	55 (19.6)	0.091^∞^
Apgar score, *n* (%)			
1st minute			
≤7	22 (95.7)	172 (61.2)	**0.001** ^∞^
≥8	1 (4.3)	109 (38.8)	
5th minute			
≤7	15 (65.2)	64 (22.8)	<0.0001*
≥8	8 (34.8)	217 (77.2)	
Respiratory distress syndrome, *n* (%)	22 (95.7)	137 (48.8)	<**0.0001** ^∞^
Surfactant administration, *n* (%)	22 (95.7)	114 (40.6)	**0.008** ^∞^
Invasive mechanical ventilation, *n* (%)	23 (100)	137 (48.8)	<**0.0001** ^∞^
Invasive mechanical ventilation, median day (min–max)	35 (4–140)	4 (1–50)	<**0.0001** ^¥^
Nosocomial sepsis, *n* (%)	15 (65.2)	59 (20.9)	<0.001*
Necrotizing enterocolitis ≥ 2A, *n* (%)	1 (4.3)	7 (2.5)	0.175^∞^
Retinopathy of prematurity ≥ 2, *n* (%)	7 (30.4)	3 (1.0)	<**0.0001** ^∞^
Intraventricular hemorrhage III-IV, *n* (%)	1 (4.3)	3 (1.0)	0.086^∞^
Cystic periventricular leukomalacia, *n* (%)	9 (39.1)	19 (6.7)	<0.0001*
Patent ductus arteriosus	5 (21.7)	14 (4.9)	0.001*
NICU stay, median day (min–max)	90 (49–147)	46 (1–160)	<**0.0001** ^¥^

*Chi-squared test, ^¥^Mann-whitney *U* test, ^∞^Fisher's exact test; NCPAP: nasal continuous positive airway pressure; NICU: neonatal intensive care unit.
